# Population Pharmacokinetics of Vancomycin in Chinese ICU Neonates: Initial Dosage Recommendations

**DOI:** 10.3389/fphar.2018.00603

**Published:** 2018-06-26

**Authors:** Zhi-ling Li, Yi-xi Liu, Zheng Jiao, Gang Qiu, Jian-quan Huang, Yu-bo Xiao, Shu-jin Wu, Chen-yu Wang, Wen-juan Hu, Hua-jun Sun

**Affiliations:** ^1^Department of Pharmacy, Shanghai Children's Hospital, Shanghai Jiao Tong University, Shanghai, China; ^2^Department of Pharmacy, Huashan Hospital, Fudan University, Shanghai, China; ^3^School of Basic Medicine and Clinical Pharmacy, China Pharmaceutical University, Nanjing, China; ^4^Department of Neonatology, Shanghai Children's Hospital, Shanghai Jiao Tong University, Shanghai, China; ^5^Department of Pharmacy, Renmin Hospital of Wuhan University, Wuhan, China; ^6^Department of Pharmacy, Gansu Provincial Hospital, Lanzhou, China

**Keywords:** neonate, vancomycin, population pharmacokinetic, Monte Carlo simulation, individualized therapy

## Abstract

The main goal of our study was to characterize the population pharmacokinetics of vancomycin in critically ill Chinese neonates to develop a pharmacokinetic model and investigate factors that have significant influences on the pharmacokinetics of vancomycin in this population. The study population consisted of 80 neonates in the neonatal intensive care unit (ICU) from which 165 trough and peak concentrations of vancomycin were obtained. Nonlinear mixed effect modeling was used to develop a population pharmacokinetic model for vancomycin. The stability and predictive ability of the final model were evaluated based on diagnostic plots, normalized prediction distribution errors and the bootstrap method. Serum creatinine (Scr) and body weight were significant covariates on the clearance of vancomycin. The average clearance was 0.309 L/h for a neonate with Scr of 23.3 μmol/L and body weight of 2.9 kg. No obvious ethnic differences in the clearance of vancomycin were found relative to the earlier studies of Caucasian neonates. Moreover, the established model indicated that in patients with a greater renal clearance status, especially Scr < 15 μmol/L, current guideline recommendations would likely not achieve therapeutic area under the concentration-time curve over 24 h/minimum inhibitory concentration (AUC_24h_/MIC) ≥ 400. The exceptions to this are British National Formulary (2016–2017), Blue Book (2016) and Neofax (2017). Recommended dose regimens for neonates with different Scr levels and postmenstrual ages were estimated based on Monte Carlo simulations and the established model. These findings will be valuable for developing individualized dosage regimens in the neonatal ICU setting.

## Introduction

After more than 60 years of widespread clinical use, vancomycin remains the gold standard antibiotic prescribed for the treatment of sepsis caused by *coagulase-negative Staphylococci* and methicillin-resistant *Staphylococcus aureus* in neonatal intensive care units (ICU) (Tong et al., 2016). In 2011, guidelines issued by the Infectious Disease Society of America and pediatric-specific guidance recommended targeting trough concentrations of >15 mg/L for critically ill children and >10 mg/L for all other pediatric patients (Liu et al., 2011). Low concentrations may result in less-effective therapy and an increased propensity for the development of bacterial resistance, whereas high concentrations are reported to be associated with nephrotoxicity and ototoxicity, although these toxic effects are less common in neonates (Anderson et al., 2007).

Vancomycin is mainly eliminated by the kidneys and is highly correlated with creatinine clearance. In neonates, the pharmacokinetics of vancomycin are highly variable because of developmental and pathophysiological changes (Stockmann et al., 2015). It is both challenging and imperative to optimize the vancomycin dosage regimen to achieve adequate exposure within a short period of time.

The maximum a *posteriori* Bayesian estimation method has already been used to support vancomycin dosing decisions in adults (Deng et al., 2013; Jacqz-Aigrain et al., 2015) and children (Le et al., 2014). To obtain accurate estimation with this method for individualized therapy, it is crucial that reliable population pharmacokinetic characteristics are known for the target patients. Several pharmacokinetic studies have been conducted in different ethnic groups of adults and showed different clearance (CL) for vancomycin both in Chinese patients [6.05 (2.38–6.06) L/h and 6.06 ± 2.46 L/h] (He et al., 2014; Lin et al., 2015) and in Caucasian patients (4.03 ± 1.7 and 5.83 ± 2.39 L/h) (Guay et al., 1993; Sánchez et al., 2010). Moreover, several population pharmacokinetic studies have been conducted in Caucasian (Seay et al., 1994; Grimsley and Thomson, 1999; Capparelli et al., 2001; Mulla and Pooboni, 2005; Allegaert et al., 2007; Anderson et al., 2007; Marques Minana et al., 2010; Oudin et al., 2011; Mehrotra et al., 2012; Zhao et al., 2013; Frymoyer et al., 2014), Japanese (Kimura, 2002), and Malaysian (Lo et al., 2010) neonates, but a large variability in clearance was observed among groups. The average clearance across all three populations ranged from 0.08 to 0.50 L/h for a neonatal patient with a weight of 3 kg and postmenstrual age (PMA) of 40 weeks.

As little information about vancomycin population pharmacokinetics is known for Chinese patients, the goal of the present study was to establish a population pharmacokinetic model for critically ill Chinese neonates receiving vancomycin therapy and to provide a rational dosage regimen for Chinese neonates.

## Methods

### Patients

Chinese neonates who received vancomycin in the neonatal ICU at Shanghai Children's Hospital (Shanghai, China) between January 2013 and December 2016 were enrolled. Patients included in this study were neonates with PMA ≤ 48 weeks for preterm neonates and with postnatal age (PNA) ≤ 28 days for term neonates. All patients were treated with vancomycin for at least 3 days, and at least one vancomycin concentration was assayed. Patients with extracorporeal membrane oxygenation or continuous renal replacement therapy were excluded from the current study. The following information was retrospectively collected from electronic medical records: gestational age (GA), PMA, PNA, body weight (WT), dosing history and concentration of vancomycin, serum creatinine (Scr) levels, clinical laboratory tests of other renal and hepatic functions, and co-administered medications.

This study was carried out in accordance with the recommendations of the Declaration of Helsinki (2000). The protocol was approved by the Ethics Committee of Shanghai Children's Hospital. Parents or guardians of patients gave their informed consent before enrollment.

The dose of vancomycin (Vancocin, Lilly, S.A, Suzhou, China) was 10–15 mg/kg, which was administrated every 8 h (q8h) or every 12 h (q12h) with a 2-h infusion, in accordance with local protocols. Blood samples were collected 1 h after completion of drug infusion (peak concentration) or half an hour before the start of vancomycin administration (trough concentration) for each patient. Trough and peak concentrations were obtained after at least four repeated doses. Potential outlier data points (observations) were identified by employing conditional weighted residual (CWRES) results outside a range of ± 6 (Byon et al., 2013).

### Bioassay

Concentrations of vancomycin were determined using the fluorescence polarization immunoassay with an ARCHITECT i2000SR (Abbott Laboratories, Chicago, IL, USA). The limit of detection was 1 mg/L, and the calibration range of this assay was 3 to 50 mg/L. The intra-day and inter-day coefficients of variation (CVs) were all < 20%.

Scr assays were performed with the enzymatic method and were analyzed with a 7180 Automatic Analyzer (Hitachi High-Tech Science Systems Corporation, Tokyo, Japan). The calibration range was from 3 to 100 mg/L. The intra-day and inter-day CVs were all < 3.75%. Creatinine can pass through the placenta and many endogenous factors can influence creatinine determination in neonates, which can limit the concentration of creatinine detected.

### Model building

Population pharmacokinetic modeling was performed using the NONMEM program (Version 7.4; Icon Inc, PA, USA) compiled with gFortran (Version 4.9.2; http://www.gfortran.org). R (Version 3.3.1; http://www.r-project.org) and the Xpose package (Version 4.5.3; http://xpose.sourceforge.net) were used for visual diagnosis. The first-order conditional estimation method with η–ε interaction (FOCE-I) was used throughout the model-building procedure (Wählby et al., 2001).

#### Base model

The vancomycin concentration data were fitted by the one or two compartment with a first-order elimination model. NONMEM subroutines were specified as ADVAN1-TRANS2 or ADVAN3-TRANS4, respectively.

An exponential model (Equation 1) was used to account for inter-individual variability (IIV):

(1) $$\begin{array}{l} {\text{P}}_{\text{i}}=\text{TV}(\text{P})\times \exp(\eta_{\text{i}}) \end{array}$$

where TV(P) is the typical value of the pharmacokinetic parameter, and P_i_ refers to the pharmacokinetic parameter of the *i*th patient with random variable η_i_, which is normally distributed with a mean of zero and variance of ω^2^.

Residual unexplained variability was tested by an additive model (Equation 2), exponential model (Equation 3), or a combined additive and exponential model (Equation 4):

(2) $$\begin{array}{l} \text{Y}=\text{IPRED}+\varepsilon \end{array}$$

(3) $$\begin{array}{l} \text{Y}=\text{IPRED}\times \exp(\varepsilon ) \end{array}$$

(4) $$\begin{array}{l} \text{Y}=\text{IPRED}\times \exp(\varepsilon_{1})+\varepsilon_{2} \end{array}$$

where Y represents the observation, IPRED is the individual predicted concentration, and ε is a symmetrically distributed variable, with a mean of zero and variance of σ^2^.

#### Covariate models

The continuous covariates, including WT, GA, PNA, PMA, Scr, blood urea nitrogen, serum albumin concentration, aspartate transaminase and alanine transaminase, and categorical covariates, including gender, concomitant drug (ceftriaxone, meropenem, gentamicin, furosemide, ibuprofen, and dexamethasone), were screened for their influence on clearance and the volume of distribution.Covariate screening was performed according to the following four steps:

**Step 1** Body weight and age have significant impacts on the pharmacokinetics of vancomycin in neonates (Wallis and Williamson, 2011; Jacqz-Aigrain et al., 2015) and physical maturation is a time-dependent characteristic that must be considered in neonates (Schmidt and Derendorf, 2014). Therefore, body weight (WT) and age (PNA, GA, and PMA) were screened first. Four different models based on allometric scaling were tested using Equation (5):
  (5) $$\begin{array}{l} {\text{P}}_{\text{i}}\text{= TV}\left(\text{P}\right)\times {\left(\frac{\text{COV}}{{\text{COV}}_{\text{median}}}\right)}^{\theta }\times \text{MF} \end{array}$$

where COV_median_ is the median of the covariate, MF is the maturation factor that is defined as the process of becoming mature. The model displaying the best fit was selected for further analysis.

*Model I*: In the simplest exponent model, the exponent θ was estimated, and MF was fixed to 1, indicating that maturation was not considered. This model is shown as Equation (6):

(6) $$\begin{array}{l} {\text{P}}_{\text{i}}\text{= TV}\left(\text{P}\right)\times {\left(\frac{\text{COV}}{{\text{COV}}_{\text{median}}}\right)}^{\theta } \end{array}$$

*Model II*: For the maturation model (Holford et al., 2013), the exponent θ was assigned a fixed value of 0.75, and MF was calculated according to Equation (7):

(7) $$\begin{array}{l} \text{MF=}\frac{1}{\text{1+}{\left(\frac{\text{Age}}{{\text{TM}}_{50}}\right)}^{\text{Hill}}} \end{array}$$

where TM_50_ is the age (in terms of GA, PMA, or PNA) at which clearance maturation reaches 50% of that of adults, and Hill is the slope parameter for the sigmoid E_max_ maturation model.

*Model III*: This is referred to as the WT-dependent exponent model (Holford et al., 2013):

(8) $$\begin{array}{l} \theta =\theta_{0}-\frac{{\text{k}}_{\max}\;\times \;{\text{WT}}^{\text{Hill}}}{{\text{k}}_{50}^{\text{Hill}}{\text{+WT}}^{\text{Hill}}} \end{array}$$

*Model IV*: This is referred to as the age-dependent exponent model (Ding et al., 2015):

(9) $$\begin{array}{l} \theta =\theta_{0}-\frac{{\text{k}}_{\max}\;\times \;{\text{Age}}^{\text{Hill}}}{{\text{k}}_{50}^{\text{Hill}}{\text{+Age}}^{\text{Hill}}} \end{array}$$

where θ_0_ is the value of the exponent at a theoretical WT of zero (Equation 8) or at birth (0 years) (Equation 9), *k*_max_ is the maximum decrease of the exponent, *k*_50_ is the WT (Equation 8) or age (Equation 9) at which a 50% decrease relative to the maximum decrease is attained, and the Hill coefficient is used to determine the steepness of the sigmoid decline.

**Step 2** In previous studies, renal function has been identified as an important covariate (Grimsley and Thomson, 1999; Capparelli et al., 2001; Kimura, 2002; Oudin et al., 2011; Mehrotra et al., 2012; Zhao et al., 2013; Derschmills et al., 2014; Frymoyer et al., 2014). We thus investigated and Scr by using the exponential model (Equation 6) and a linear model (Equation 10):
  (10) $$\begin{array}{l} {\text{P}}_{\text{i}}\text{=TV}\left(\text{P}\right)\text{+}\theta \times ({\text{COV-COV}}_{\text{median}}) \end{array}$$

The one displaying the best fit was used for further analysis.

**Step 3**. The remaining covariates were then accessed sequentially by forward inclusion and backward elimination approaches using the exponential model (Equation 8) or linear model (Equation 10) for continuous variables and a proportional model (Equation 11) for categorical variables, such as gender in the P_i_ of vancomycin:
  (11) $$\begin{array}{l} {\text{P}}_{\text{i}}=\{\begin{array}{c} \text{TV}\left(\text{P}\right)\text{ if male }\\ \text{TV}\left(\text{P}\right)\times \theta \text{ if female} \end{array} \end{array}$$

Where θ is the fractional change in TV(P) for males.

**Step 4** Taking into account the rapid variation in the physical status of neonates, the covariates identified above could be defined as time-varying covariates to illustrate IIV. The time-varying covariates model splits the individual covariate effects into baseline and change-from-baseline effects. Two different models based on covariate scaling of the pharmacokinetic parameters were tested using Equations (12) and (13):
  (12) $$\begin{array}{l} {\text{P}}_{\text{i}}=\text{TV}\left(\text{P}\right)\times [1+\;\theta_{\text{BCOV}}\times \left({\text{BCOV-BCOV}}_{\text{median}}\right)\\ \;+\theta_{\text{DCOV}}\times \text{DCOV}]\times \exp\left(\;\eta ,\text{Pi}\right) \end{array}$$
  (13) $$\begin{array}{l} {\text{P}}_{\text{i}}=\text{TV}\left(\text{P}\right)\times [1+\;\theta_{\text{COV}}\times \exp(\eta_{\text{COV}},{\text{P}}_{\text{i}})\times \text{COV}\\ \;-{\text{COV}}_{\text{median}})]\times \exp\left(\eta ,\text{Pi}\right) \end{array}$$

where BCOV is the baseline value of the covariate; BCOV_median_ is the median of the baseline value of covariate; θ_BCOV_ describes the effect of IIV. DCOV is equal to the current covariate value minus BCOV at each time point and corresponds to the fractional change in the typical value with each unit difference in BCOV relative to BCOV_median._ θ_DCOV_ describes the effect of covariate variation within an individual and is the fractional change in the typical value with individual changes in COV, and (η, P_i_) refers to variable with a mean of zero and variance of ω^2^ that describes the random effect of P_i_.

If θ_BCOV_ and θ_DCOV_ are different, DCOV is fixed to zero, and an additional variance parameter (η_COV_, P_i_) that accounts for IIV to influence the covariates for the population parameter estimates is included (Equation 14).

θ_COV_ is the parameter estimation value of the covariate, COV is the value of the covariate, COV_median_ is the median of the covariate, η_COV_ is random variable (with zero mean and variance ω^2^).

#### Model selection criteria

Structural models were selected through Akaike information criteria (AIC) and Bayesian information criteria (BIC) calculated using Pirana software (version 2.9.0, http://www.pirana-software.com/) (Keizer et al., 2011) Models with lower AIC and BIC values were considered superior (Byon et al., 2013).

Nested models in covariate screening were compared statistically using a likelihood ratio test on the differences in the objective function value (OFV). The change was considered significant if the decrease in OFV was >3.83 (χ^2^*, df* = 1, *P* < 0.05) for the forward inclusion step, and the increase in OFV was >6.63 (χ^2^*, df* = 1, *P* < 0.01) for the backward elimination step.

The covariates in the model were selected based on physiological plausibility of parameter estimates, goodness-of-fit plots, and statistical significance.

### Model evaluation

The performance of the final model was first evaluated by visual inspection of diagnostic goodness-of-fit plots. Goodness-of-fit plots included the following scatterplots: observation (DV) vs. individual prediction (IPRED), DV vs. population prediction (PRED), conditional weighted residual errors (CWRES) vs. time, and CWRES vs. PRED (Hooker et al., 2007).

The robustness of the model was assessed using a nonparametric bootstrap (Ette, 1997), with repetition of 2000 NONMEM runs of the final model. The bootstrap median parameter values and the percentile bootstrap 95% confidence intervals were compared with the respective values estimated from the final model.

Normalized prediction distribution error (NPDE) (Comets et al., 2008) was used to evaluate the predictive performance of the model based on a Monte Carlo simulation with the R package (version 2.0, http://www.npde.biostat.fr/). NPDE results were summarized graphically using (1) quantile-quantile plot of the NPDE, (2) a histogram of the NPDE, (3) scatterplot of NPDE vs. time, and (4) scatterplot of NPDE vs. PRED. If the predictive performance is satisfied, the NPDE will follow a normal distribution (Shapiro-Wilk test) with a mean value of zero (*t*-test) and a variance of one (Fisher's test).

### Dosing regimen design

The final established population pharmacokinetic model was used to obtain dosing regimens for vancomycin to reach AUC_24h_/MIC ≥ 400 which is known to produce an effective therapeutic outcome (Jacqz-Aigrain et al., 2015). When MIC = 1 mg/L, the daily dose can be calculated by the final model for determining the clearance and Equation 14:

(14) $$\begin{array}{l} \text{Dose }\left(\text{mg/day}\right)\text{=400}\times \text{CL} \end{array}$$

Simulations were performed for virtual patients with various levels of renal function and ages, to determine the most appropriate scheme to satisfy the therapeutic criteria. For this purpose, 1,000 replicates of each scenario were simulated by the Monte Carlo method and were completed by the $SIMULATION modules in NONMEM software.

Virtual patients were designated as having PMAs covering a 2-week window between 28 and 44 weeks, and their corresponding WTs were calculated according to the World Health Organization growth chart for infants (Centers for Disease Control Prevention, 2009). Patients were designated a Scr level of 15, 20, or 35 μmol/L and with an age of PNA 7 days (≤1 week) or PNA 15 days (>2 weeks).

Vancomycin dosage recommendations are highly variable as illustrated by differences in various guidelines (Jacqz-Aigrain et al., 2015). The dose regimens were then compared to the guidelines used in different regions or medical centers, including the FDA's labeled dosage regimen, British National Formulary (BNF) for Children 2016–2017 and Blue Book 2016 from the UK, Neonatal Formulary 2015 from the Europe, Red Book 2015, Pediatric & Neonatal Dosage Handbook 2015–2016, and Neofax 2017 (http://neofax.micromedexsolutions.com/neofax/neofax.php) from the US.

## Results

### Patients

Data from 165 vancomycin measurements, with a trough concentration for 75 of the measurements and a peak concentration for 90 of the measurements, from 80 subjects were available to perform the population pharmacokinetics analysis. For each subject, an average of two samples were obtained. The GA range was 25.7 weeks to 41.1 weeks, with a mean WT of 2.87 kg. Of these patients, 59% were preterm infants and 57% had respiratory tract infections. All the observation were included, and no outlier records were identified. Clinical characteristics of the neonates included in the analysis are summarized in Table 1.

**Table 1:** Demographic and clinical data for neonates in this study.

**Variable**	**Modeling group**	**Median (range)**
Number of patients (male/female)	80 (54/26)	/
Number of observations (trough /peak)	165 (75/90)	/
Postnatal age (PNA), days	32.3 ± 24.1	24 (4–126)
Gestational weeks (GA), weeks	34.7 ± 4.31	34 (25.7–41.1)
Postmenstrual age (PMA), weeks	39.4 ± 3.60	40.0 (29–47.1)
Weight, kg	2.87 ± 0.89	2.74 (1.4–5.6)
Height, cm	46.8 ± 4.72	47 (37–65)
Serum creatinine, μmol/L	23.2 ± 10.4	28.3 (5.85–61.6)
Blood urea nitrogen, mmol/L	4.96 ± 3.89	4.1 (0.4–28.5)
Total protein, g/L	48.6 ± 7.38	48.2 (33–67.6)
Albumin, g/L	32.4 ± 5.49	32 (21.6–46.8)
Aspartate aminotransferase, U/L	43.6 ± 85.1	18 (3–575)
Glutamic-pyruvic transaminase, U/L	77.6 ± 109	41 (9-696)
Dosage, mg	45 ± 16.4	42 (20–105)
Trough concentration, mg/L	11.2 ± 7.92	9.15 (3.14–42.9)
Peak concentration, mg/L	22.3 ± 11.0	20.3 (4.09–51.9)

### Model building

A one-compartment model with first order elimination described the pharmacokinetic of vancomycin. The residual unexplained variability was described best by a proportional model. As only peak and trough samples were collected, the relative standard error (RSE) of the IIV of the volume of distribution was poor (>50%) and was not estimated.

For the first step of covariate screening, several models were tested, and the results are shown in Table 2. Among the four models examined, the simple exponent model (Model I) and maturation model (Model II) had lower Akaike information criteria and Bayesian information criteria than the ADE model (Model III) and BDE model (Model IV). The maturation model had a condition value of 23,082, much greater than 1,000, indicating model instability (Byon et al., 2013). Moreover, the RSE of most PK parameters in the maturation model were more than 100%, implying inaccuracy of the model parameter estimates. Therefore, the simple exponent model was employed in further analyses.

**Table 2:** Population pharmacokinetic estimates of vancomycin of maturation model.

**Parameters**	**Model I**	**Model II**	**Model III**	**Model IV**
**Model description**	**CLp**×**(WT/WT**_median_**)** *^*k*^*×**MF**			
MF	1	$\text{MF}=\frac{1}{1+{\left(\frac{\text{Age}}{\text{T}{\text{M}}_{50}}\right)}^{\text{Hill}}}$	1	1
*k*	/	0.75	$\theta_{0}-\frac{{\text{k}}_{\max}\times \text{W}{\text{T}}^{\text{Hill}}}{{{\text{k}}_{50}}^{\text{Hill}}+\text{W}{\text{T}}^{\text{Hill}}}$	$\theta_{0}-\frac{{\text{k}}_{\max}\times \text{Ag}{\text{e}}^{\text{Hill}}}{{{\text{k}}_{50}}^{\text{Hill}}+{\text{Age}}^{\text{Hill}}}$
Objective function value	855.1	852.2	871.1	871.1
Akaike information criteria	867.1	864.2	895.9	895.9
Bayesian information criteria	885.7	882.8	855.1	855.1
Condition number	4.54	23082	510000	641732
CLp (RSE%)	0.319 (5.1%)	0.911 (235%)	0.319 (5.1%)	0.319 (5.1%)
*k* (RSE%)	1.57 (10.9%)	/	/	/
TM_50_ (RSE%)	/	46.9 (102%)	/	/
Hill (RSE%)	/	4.45 (105%)	23.6 (17.2%)	60.7 (23.4%)
θ_0_ (RSE%)	/	/	1.57 (10.9%)	1.57 (10.9%)
k_max_ (RSE%)	/	/	1.08 (25%)	1.08 (25%)
k_50_ (RSE%)	/	/	12.7 (53.1%)	62.3 (22%)
**INTER-INDIVIDUAL VARIABILITY**				
CL (%CV)	38.6% (28.9%)	39.2% (26.4%)	39.2% (26.4%)	39.2% (26.4%)
**RESIDUAL VARIABILITY**				
Proportional (%CV)	37.9% (19.2%)	37.9% (20.4%)	37.9% (20.4%)	37.9% (20.4%)

*CL_p_ typical value of apparent clearance (L/h), Hill coefficient determining the steepness of the sigmoidal decline, k the exponents for body weight, k_max_, maximum decrease, k_50_ the body weight or age at which there is a 50 % decrease in the k_max_, MF maturation function, RSE: relative standard error, TM_50_ the age at which clearance maturation reaches 50% of that of adults, θ_0_ the exponent at a theoretical WT of 0 kg or age at birth*.

Second, Scr and WT were identified as significant covariates and were thus retained in the model. Further incorporation of time-varying covariates did not improve the model performance, which could be attributed to the short treatment duration of vancomycin.

The final model for vancomycin clearance was represented by Equation 15, and WT was added to volume of distribution for physiologic plausibility as shown in Equation 16.

(15) $$\begin{array}{l} \text{CL}(\text{L/h})=0.309\times {\left[\frac{\text{WT}\left(\text{kg}\right)}{2.9}\right]}^{1.55}\times {\left[\frac{23.3}{\text{Scr}\left(\mu \text{mol/L}\right)}\right]}^{0.337} \end{array}$$

(16) $$\begin{array}{l} \text{V}\left(\text{L}\right)=2.63\times {\left[\frac{\text{WT}\left(\text{kg}\right)}{2.9}\right]}^{1.05} \end{array}$$

where CL is vancomycin clearance, WT is current body weight in neonates, Scr is serum creatinine, V is volume of distribution for vancomycin. The final model parameter estimates are shown in Table 3.

**Table 3:** Population pharmacokinetic estimates of vancomycin of final model and Bootstrap evaluation.

**Parameter**	**NONMEM**		**Bootstrap**		**Bias**
	**Estimate**	**RSE (%)**	**Median**	**2.5% ~97.5%**	
**STRUCTURE MODEL PARAMETER**					
θ_1_	0.309	5	0.308	0.276–0.339	−0.36%
θ_2_	1.55	10	1.55	1.21–1.88	0.19%
θ_3_	0.337	40	0.342	0.09–0.61	1.86%
θ_4_	2.63	8	2.62	2.18–3.11	−0.02%
θ_5_	1.05	27	1.06	0.47–1.59	−0.25%
**INTER-INDIVIDUAL VARIABILITY**					
CL (%CV)	37.9%	26	36.7%	25.2–46.4%	−21.6%
**RESIDUAL VARIABILITY**					
Proportional (%CV)	37.5%	19	36.8%	30.3–44.2%	−14.8%

*Final model: $CL\left(L/h\right)=\theta_{1}\times {\left[\frac{WT\left(kg\right)}{2.9}\right]}^{\theta_{2}}\times {\left[\frac{23.3}{Scr\left(\mu mol/L\right)}\right]}^{\theta_{3}}$*.

*$V\left(L\right)=\theta_{4}{\times \left[\frac{WT\left(kg\right)}{2.9}\right]}^{\theta_{5}}$*.

*CL clearance (L/h), V volume of distribution (L), WT body weight (kg), Scr serum creatinine (μmol/L); NONMEM: nonlinear mixed-effects model; %RSE: relative standard error (standard error/estimate × 100%); Bias%: relative bias of estimates by NONMEM to the median estimates by 2,000 bootstrap procedures, Bias% = (Bootstrap Median—NONMEM estimate)/ NONMEM estimate × 100%*.

### Model evaluation

**Figure 1:**
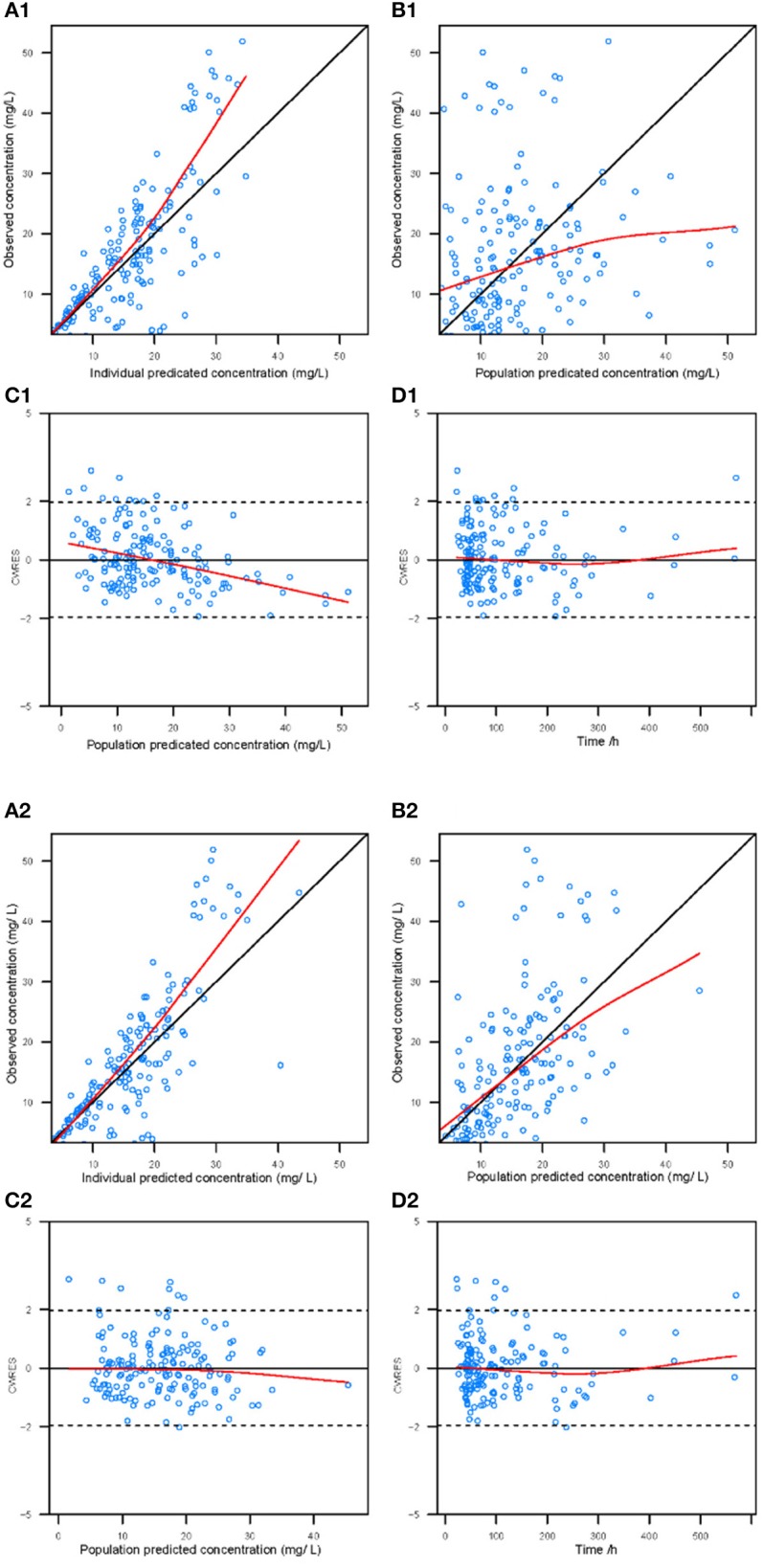
Diagnostic goodness-of-fit plots for the base model (1) and the final model (2). **(A)** The individual predicted concentration (IPRED) vs. the observed concentration. **(B)** The population predicted concentration (PRED) vs. the observed concentration. **(C)** The PRED vs. the conditional weighted residual errors (CWRES). **(D)** The time after dose vs. CWRES. The black solid lines are the reference lines, and red solid lines are the loess smooth lines.

Goodness-of-fit plots for the base model and final model are shown in Figure 1. Compared with the base model, the final model showed no obvious bias or significant trends within these scatterplots. Moreover, the data fitting for the final model was much improved relative to that of the base model.

The results from the bootstrap procedure are shown in Table 3. The median values from the bootstrap procedure were close to the parameter estimates from the NONMEM, with <5% bias. In addition, more than 99% of the bootstrap runs were successful, indicating that the model was stable.

**Figure 2:**
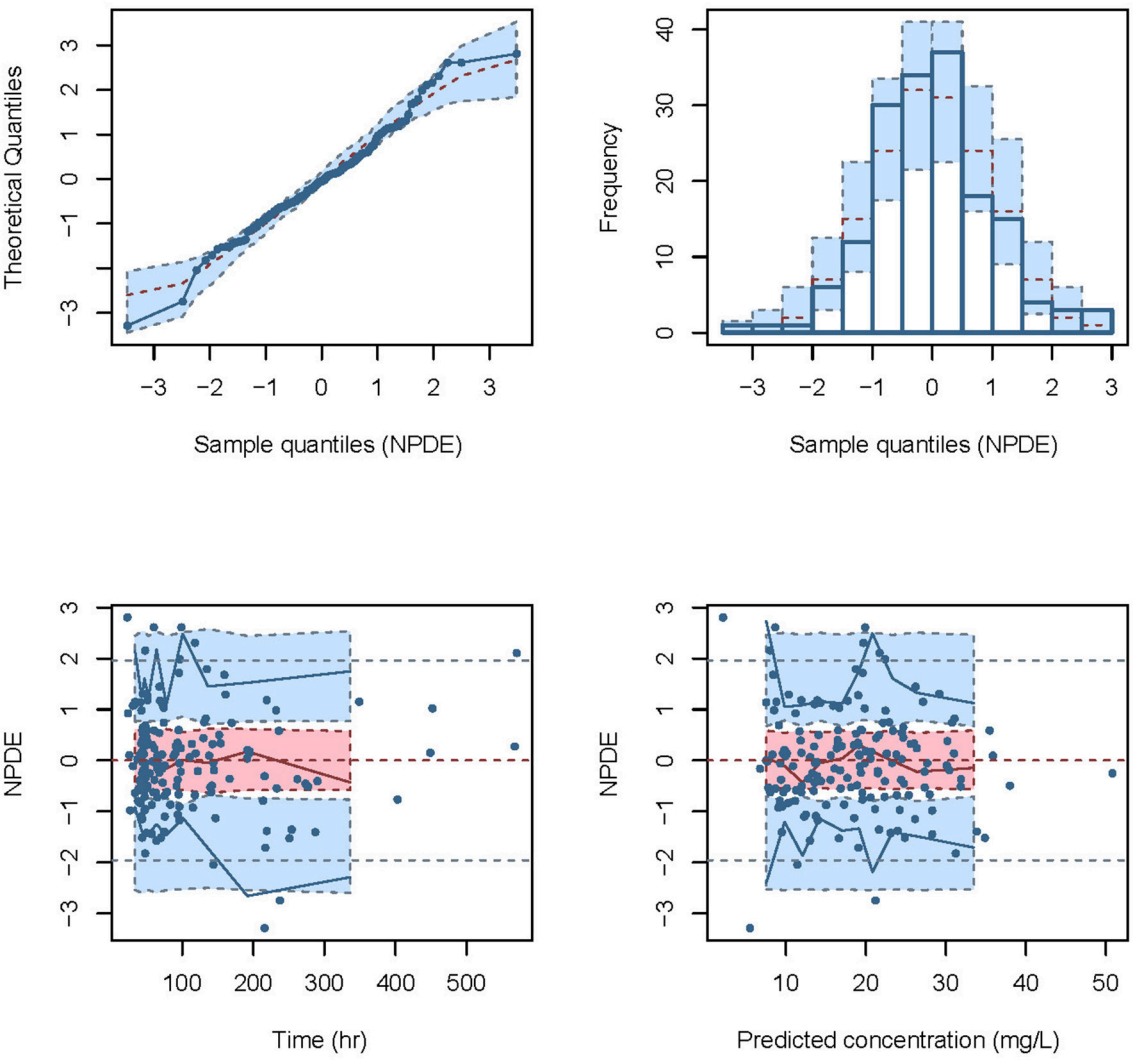
Normalized prediction distribution error (NPDE) for the final model. Quantile-quantile plots of NPDE vs. the expected standard normal distribution (upper left). Histogram of NPDE values with the standard normal distribution overlayed (upper right). Scatter plot of the time vs. NPDE (lower left). Scatterplot of predictions vs. NPDE (lower right).

The NPDE distribution and histogram are presented in Figure 2. The assumption of a normal distribution for the differences between predictions and observations was acceptable. The quantile-quantile plots and histogram also confirmed the normality of the NPDE (Figure 2).

### Dosing regimen design

**Figure 3:**
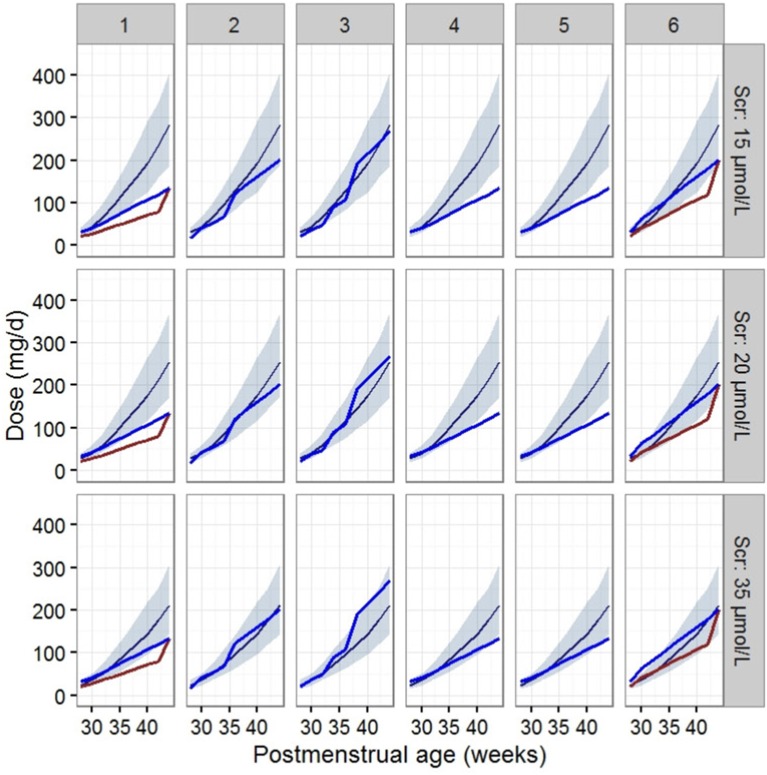
Vancomycin dosage regimen recommended by the six guidelines relative to the regimen recommended by our final model in typical patients when AUC_24h_/MIC ≥ 400. The six guides correspond to (1) the FDA labeled dosage (2017), (2) the British National Formulary (2016–2017) and the Blue Book (2016), (3) the Neonatal Formulary (2015), (4) the Red Book (2015), (5) the Pediatric and Neonatal Dosage Handbook (2015-2016), and (6) Neofax (2017). The red and blue lines in (1) and (6) refer to dosage guidelines for patients with a PNA of 7 days and 15 days respectively. The blue lines in (2),(3),(4), and (5) refers to dosage for PNA of both 7 and 15 days. The dark blue smooth curve represents the mean dosage for the present study, and the light blue ribbon corresponds to the 15–85% dosage interval.

The dosage regimens recommended by current guidelines and estimated by the established model are displayed in Figure 3 and Table 4. The guideline schemes from the BNF for children, the Blue Book and the Neonatal Formulary are nearly in accordance with the 15% to 85% dosage interval from the present model that achieves a therapeutic target of 400 ≤ AUC_24h_/MIC <800. The targeted vancomycin concentration based on the FDA labeled dosage, the Red Book, the Pediatric & Neonatal Dosage Handbook, and Neofax was not likely sufficient, especially for neonates with Scr of 15 μmol/L. Moreover, Neonatal Formulary shows an overdose for a subpopulation of neonates with Scr of 35 μmol/L, which might indicate an increased risk of toxicity.

**Table 4:** Dosage recommendations based on the final model.

**Weight (kg)**	**PMA (weeks)**	**Serum Creatitine (μmol/L)**	**Dosage recommendations Dose (mg/kg)**
1.0-1.49	28–30	10	15–20 mg/kg every 12 h
		25	12.5–15 mg/kg every 12 h
		45	15–17.5 mg/kg every 18 h
		60	12.5–15 mg/kg every 18 h
1.5–2.49	31–34	10	12.5–17.5 mg/kg every 8 h
		25	15–20 mg/kg every 12 h
		45	12.5–15 mg/kg every 12 h
		60	10–12.5 mg/kg every 12 h
2.5–3.49	35–38	10	17.5–20 mg/kg every 8 h
		25	12.5–15 mg/kg every 8 h
		45	17.5–20 mg/kg every 12 h
		60	15–17.5 mg/kg every 12 h
3.5–4.49	39–42	10	15–20 mg/kg every 6 h
		25	10–12.5 mg/kg every 6 h
		45	12.5–15 mg/kg every 8 h
		60	10–12.5 mg/kg every 8 h
4.5–5.5	43–45	10	17.5–20 mg/kg every 6 h
		25	12.5–15 mg/kg every 6 h
		45	12.5 mg/kg every 6 h
		60	10 mg/kg every 6 h

## Discussion

In the present study, we first developed a population pharmacokinetic model for vancomycin in Chinese neonates in the ICU and found that WT and Scr levels have significant influences on clearance. Little obvious ethnic difference of vancomycin clearance was shown in Asian and Caucasian neonates from our study.

Differences in vancomycin pharmacokinetics have been noted between Asian and Caucasian populations based on two recently published studies (Lin et al., 2015, 2016). To expand this analysis, we first looked at a group of 12 previous studies that examined neonatal populations from various countries. The population within some of these studies were heterogeneous. Patients with extracorporeal membrane oxygenation were included in the model of Mulla et al. (Mulla and Pooboni, 2005), and the majority of neonates used to develop the models of Allegaert et al. (2007) and Capparelli et al. (2001) were preterm. Due to differences in the physiologic development of neonates within these populations, models from these three studies were excluded from the comparison. Additionally, neonates within 6 other studies (Seay et al., 1994; Grimsley and Thomson, 1999; Lo et al., 2010; Marques Minana et al., 2010; Oudin et al., 2011; Zhao et al., 2013) were smaller, with a mean WT <2 kg. Given the relative weight of neonates in our study and the impact of WT on vancomycin pharmacokinetic parameters, these six studies were excluded from comparisons. The three remaining studies (Kimura, 2002; Mehrotra et al., 2012; Frymoyer et al., 2014) were included in our analysis of ethnic differences regarding vancomycin clearance.

The clearance of a standardized patient, as determined by the different models, was calculated to investigate differences in vancomycin clearance relative to ethnicity. The standardized patient had a WT of 2.8 kg, PMA of 37 weeks with different Scr levels.

As shown in Table 5, across varying Scr levels ranging from 20 μmol/L to 50 μmol/L. there was 27~39 % difference in vancomycin clearance between the current study and the study by Mehrotra (Mehrotra et al., 2012), but comparable to another study (<13%) which was also conducted in US (Frymoyer et al., 2014). This information does not support the conclusion that there are obvious differences in vancomycin clearance between Chinese and Caucasians. However, the estimated clearance was much higher than the study conducted in 19 Japanese neonates (Kimura, 2002). The reason was unclear. The present study might be under-power to conclure ethnic impact on vancomycin PK in neonates. Factors in the current study, including analytical methods, and disease progression could affect the assessment of ethnic differences.

**Table 5:** Estimates of vancomycin clearance among different ethnic.

**Model**	**Country**	**Patients (samples)**	**Weight, kg median (range)**	**PNA, days median (range)**	**GA, weeks median (range)**	**Scr**, μ**mol/L median (range)**	^*^Relative differences with different Scr		
							**15** μ**mol/L**	**25** μ**mol/L**	**35** μ**mol/L**
Kimura, 2002	Japan	19 (88)	NA (0.7–5.2)	NA (3–71)	34 (24–41)	NA (17.7–79.6)	−12%	−38%	−50%
Mehrotra et al., 2012	USA	134 (267)	2.5 (0.6–5.3)	NA (1–121)	NA (25–44)	53.1 (17.7–221)	−18%	−25%	−31%
Frymoyer et al., 2014	USA	240 (1702)	2.9 (0.5–6.3)	19 (0–173)	34 (22–42)	NA (26.5–53)	NA	−9%	−7%
Present study	China	80 (165)	2.8 (1.4–5.6)	24 (4–126)	34 (26–41)	28.3 (5.9–61.6)	/	/	/

* *Relative differences = (CL–CL _present__study_)/CL _present__study_ × 100%, CL refers to estimate vancomycin clearance, CL _present__study_ refers to vancomycin clearance of present study for the typical patient of 2.8 kg, PMA of 37 weeks with different serum creatinine level, 15, 25 and 35 μmol/L refer to at the 10, 50, and 90th percentiles from our patients' Scr values. NA represents not appropriate to be estimated*.

Dosage recommendations by the label and reference books are variable as shown in Table 6. The variabilities were attributed to the different covariates considered within these recommendations. Body weight is the most notable covariate for vancomycin dosing found within all references. Age-based (as PMA, PNA, and GA) dosing is also included in most of the references, such as the FDA label recommendation, BNF for children, Blue Book, Neonatal Formulary, Pediatric and Neonatal Dosage Handbook and Neofax. Dosing based on Scr is only included in 2 references, the Red Book, and Pediatric & Neonatal Dosage Handbook and only covered Scr level > 61.9 μmol/L. However, Scr was identified to have large impacts on the CL of vancomycin in all previous population pharmacokinetic studies as well as the present study.

**Table 6:** Dosage recommendations for vancomycin in neonates^a^.

**Guide**	**Age (years) /Scr (**μ**mol/L)**	**Dosage recommendations**
Neonatal Formulary	PMA <29	20 mg/kg every 24 h
(2015)	PMA 30–33	20 mg/kg every 18 h
	PMA 34–37	20 mg/kg every 12 h
	PMA 38–44	15 mg/kg every 8 h
	PMA >45	10 mg/kg every 6 h
Red Book	Scr < 61.9	15 mg/kg every 12 h
(2015)	Scr 61.9–79.6	20 mg/kg every 24 h
	Scr 88.4–106.1	15 mg/kg every 24 h
	Scr 114.9–141.5	10 mg/kg every 24 h
	Scr> 141.5	15 mg/kg every 48 h
Pediatric Neonatal Dosage Handbook	GA ≤ 28 and Scr < 44.2	15 mg/kg every 12 h
(2015–2016)	Scr 44.2–61.9	20 mg/kg every 24 h
	Scr 70.7–97.3	15 mg/kg every 24 h
	Scr 97.3–123.8	10 mg/kgevery 24 h
	Scr> 123.8	15 mg/kgevery 48 h
	GA > 28 and Scr < 61.9	15 mg/kg every 12 h
	Scr 61.9–79.6	20 mg/kg every 24 h
	Scr 88.4–106.1	15 mg/kg every 24 h
	Scr 114.9–141.5	10 mg/kg every 24 h
	Scr 141.5	15 mg/kg every 48 h
	PNA < 7 and WT < 1200 g	15 mg/kg every 24 h
	WT 1200–2000 g	10–15 mg/kg every 12–18 h
	WT > 2000g	10–15 mg/kg every 8–12 h
	PNA ≥ 7 and WT < 1200 g	15 mg/kg every 24 h
	WT 1200–2000 g	10–15 mg/kg every 8–12 h
	WT > 2000 g	15 mg/kg every 6–8 h
BNF for children (2016–2017) and Blue Book (2016)	PMA < 29 PMA 29–35 PMA >35	15 mg/kg every 24 h 15 mg/kg every 12 h 15 mg/kg every 8 h
Neofax (2017)	PMA ≤ 29 and PNA 0–14 PNA >14	10–15 mg/kg every 18 h 10–15 mg/kg every 12 h
	PMA 30–36 and PNA 0–14 PNA >14	10–15 mg/kg every 12 h 10–15 mg/kg every 8 h
	PMA 37–44 and PNA 0–14 PNA >14	10–15 mg/kg every 12 h 10–15 mg/kg every 8 h
	PMA > 45	10–15 mg/kg every 6 h
FDA labeled dosage (2017)	PNA ≤ 7	LD: 15 mg/kg MD: 10 mg/kg every 12 h
	PNA >7 and ≤ 30	LD: 15 mg/kg MD: 10 mg/kg every 8 h

*Recommendations were selected to illustrate that dosage recommendations were highly variable, based on the covariates PNA, PMA, GA, and weight*.

*1. Neonatal Formulary: Drug use in pregnancy and the first year of life. Books Wiley-Blackwell 2015. BMJ*.

*2. Red Book: Report of the Committee on infectious diseases. Elk Grove Village. American Academy of Pediatrics, 2015*.

*3. Pediatric and Neonatal Dosage Handbook. Lexi-Comp Inc., 2015–2016*.

*4. BNF for children: The Royal Pharmaceutical Society of Great Britain, 2016–2017 and Blue book: European Society of Paediatric Infectious Diseases and the Royal College of Paediatrics and Child Health, 2016*.

*5. Neofax (http://neofax.micromedexsolutions.com/neofax/neofax.php), 2017*.

*6. FDA Labels, https://www.accessdata.fda.gov/drugsatfda_docs/label/2017/060180s047lbl.pdf, 2017*.

a *GA, Gestational age (in weeks); LD, Loading dose; MD, Maintenance dose; PMA, Postmenstrual age (in weeks); PNA, Postnatal age (in days); Scr (in μmol/L)*.

Based off data from our final model, we found that the current recommended doses of vancomycin from FDA labeled dosage, Red Book, Pediatric & Neonatal Dosage Handbook, and Neofax may be inadequate to meet a treatment target of AUC_24h_/MIC ≥ 400, especially for patients with a greater renal clearance status, especially Scr < 15 μmol/L.

This finding is consistent with several previous studies (Krivoy et al., 1998; Liu et al., 2011; Abdel et al., 2015; Zhao et al., 2015). They found that the usual recommended dose of 60 mg/kg/day did not achieve vancomycin pharmacodynamic targets in most patients. Silva, D C et al (Silva et al., 2012) reported that a vancomycin dose of 81 mg/kg/day was required to achieve an AUC_24h_/MIC > 400 in 56% of patients. Doses as high as 120 mg/kg/day were also recommended to improve the therapeutic pharmacodynamic targets (Abdel et al., 2015). Higher than usual vancomycin doses may be required to treat patients with severe Gram-positive infections.

There are several limitations to this study. The current guidelines or consensus for therapeutic drug management (TDM) of vancomycin in the United States (Rybak et al., 2009), Japan (Matsumoto et al., 2013) and China (Ye et al., 2016) recommends that trough concentrations should be collected at regular intervals in clinical settings. Therefore, only peak and trough concentrations were collected in our study and a one compartment model was applied for the structural model even though vancomycin is more normally modeled with a two-compartment model. The simplification to a one compartment model may lead to deviation of clearance estimation. However, the bias usually is <20% and does not obviously affect the estimation of the area under the curve (AUC). (Ling et al., 2014) (Kowalski and Hutmacher, 2001). The recommended regimen was based on a study population from our hospital. Therefore, generalization to other ICU neonates treated with vancomycin, especially if their covariate characteristics lie outside the range of our study population, would require additional investigations.

Moreover, several cofactors may affect the clinical outcomes of patients, such as disease progression, baseline weight, gestational age, and medication interactions. Therefore, with a limited number of patients enrolled in the study, we did not compare the clinical outcomes between those who had different exposure levels. Furthermore, as a pharmacodynamics indicator, the MIC value was obtained for only a few patients, such that we could not build a population pharmacokinetic-pharmacodynamics model, which would have been a better predictor of vancomycin's therapeutic effect.

This study offers initial vancomycin dosing regimen with varying degrees of PMA, WT, and serum creatinine for neonates. For patients with complex disease conditions, Bayesian approaches might be used to provide individualized dose recommendations instead of look-up tables or nomograms. Dose calculators and other decision support tools based on population pharmacokinetic (PPK) models (Fuchs et al., 2013) could contribute to simplifying the complex Bayes calculations and making them more intuitive to the user in clinical practice.

Recently, a nonparametric (NP) population modeling approach was reported to have advantages in patient's individual dosing adjustment, which permits development of dosage regimens to hit desired therapeutic targets with maximum precision (Jelliffe et al., 2011; Neely et al., 2018). This will be further investigated.

## Conclusion

In summary, this study has built a population pharmacokinetic model of vancomycin for Chinese neonates. WT and Scr levels were the important covariates, which affect clearance. Moreover, this study found no obvious differences in the clearance of vancomycin comparing Caucasian and Chinese neonates. For patients with normal renal function, the dosing recommendations are likely not sufficient based on the target of AUC_24h_/MIC ≥400, with the exception of British National Formulary (2016–2017), Blue Book (2016) and Neofax (2017). However, these sources provide little information on dosing adjustments based on patient renal function, thus our model provides a method for adjusting the vancomycin dose accordingly.

## Author contributions

ZL, ZJ, and HS conceived and designed the study. YL, SW, CW, HS and GQ collected the information of the neonates. YL, JH, YX, and WH performed the experiments. ZL, ZJ, and YL wrote the paper. YL, SW, YX and CW built the model and evaluated. ZL, YL, and ZJ reviewed and edited the manuscript. All authors read and approved the manuscript.

### Conflict of interest statement

The authors declare that the research was conducted in the absence of any commercial or financial relationships that could be construed as a potential conflict of interest.
